# 

**Published:** 1885-10

**Authors:** 


### BOOKS AND PAMPHLETS RECEIVED.

Studies from the Biological Laboratory, Johns Hopkins University. Vol. III., No. 3.

Constitutional Treatment of Caries and Necrosis. By Hal. C. Wyman, M.D. Detroit.

University of Pennsylvania, Catalogue and Announcement, 1884-5, Department of Biology. Philadelphia.

University of Michigan. Annual Announcement of the Department of Medicine and Surgery, 1885-6. Ann Arbor.

Cooper Medical College. Annnal Announcement, 1885. San Francisco.

Indiana State Board of Agriculture. 34th Annual Report. Vol. xxvi. Indianapolis.

Shadows on the Ethics of the International Congress. By Levi Cooper Lane, M.D. San Francisco. 1885.

Duty of the State towards the Medical Profession. An Address Delivered before the Medical Alumni Association of the University of Michigan, by Conrad George, M.D.

Tabular Statistics of One Hundred Cases of Urethral Stricture Treated by Electrolysis. By Robert Newman, M.D. New York.

Johns Hopkins University Circulars.

Annals et Bulletin de la Société de Medecine de Gand.

Quarterly Compendium of Medical Science. Philadelphia.

Jahresbericht der Koniglichen Thierarzeneischule zu Hannover Herausgegeben von dem Lehrer-Collegium Redigirt von dem Director Medicinalrath, Dr. Dammann. Siebzehnter Bericht, 1884-5.

